# Albumin/fibrinogen ratio, a predictor of chemotherapy resistance and prognostic factor for advanced gastric cancer patients following radical gastrectomy

**DOI:** 10.1186/s12893-022-01657-1

**Published:** 2022-05-28

**Authors:** Guojun Zhao

**Affiliations:** Department of Oncology, Taizhou People’s Hospital, No. 399 Hailing South Road, Taizhou, Jiangsu 225300 China

**Keywords:** Gastric cancer, Adjuvant chemotherapy, Chemotherapy resistance, Prognosis, albumin/fibrinogen ratio

## Abstract

**Background:**

The objective of this study was to investigate potential predictors of chemotherapy resistance in patients with advanced gastric cancer (GC) following radical gastrectomy.

**Methods:**

Eligible stage II/III GC patients with adjuvant chemotherapy after radical gastrectomy were enrolled in this study. A receiver operating characteristic (ROC) curve analysis was performed to assess the predictive and optimal cut-off values of continuous variables for chemotherapy resistance. Potential risk factors for chemotherapy resistance were determined with binary univariate and multivariate analyses. Potential prognostic factors for overall survival (OS) were determined by COX regression analysis. The association between survival and AFR level was examined using the Kaplan–Meier curve analysis.

**Results:**

A total of 160 patients were included in the data analysis, and 41 patients achieved chemotherapeutic resistance with an incidence of 25.6%. Pretreatment albumin/fibrinogen ratio (AFR) (cut-off value: 10.85, AUC: 0.713, P < 0.001) was a predictor for chemotherapeutic resistance by ROC curve analysis. Low AFR (< 10.85) was an independent risk factor of chemotherapeutic resistance as determined by the univariate and multivariate logistic regression analyses (OR: 2.55, 95%CI: 1.21–4.95, P = 0.005). Multivariate COX regression analyses indicated low AFR as a prognostic factor for 5-year OS (HR: 0.36, 95%CI: 0.15–0.73, P = 0.011). Low AFR was associated with poorer 5-year disease-free survival and overall survival.

**Conclusions:**

This study indicated that a low level of pretreatment AFR could serve as an independent predictor of chemotherapy resistance and postoperative prognosis in GC patients following radical gastrectomy.

## Introduction

Globally, gastric cancer (GC) has the fourth incidence and third mortality rate [1]. As the third most common cancer in China, advanced GC is associated with a poor prognosis and relatively limited treatment options [1]. Although surgical resection remains the mainstay treatment for GC, the high postoperative recurrence incidence is a great threat to GC patients [2]. Thus, postoperative adjuvant chemotherapy is a quite important supplementary treatment [3]. In Asia, curative resection with D2 lymphadenectomy in combination with postoperative adjuvant chemotherapy remains the standard treatment option for stage II/III GC [4]. However, the clinical response to chemotherapy varies remarkably among advanced GC individuals, which may greatly influence the long-term prognosis [5]. Thus, there is an urgent need to explore potential predictors of chemotherapy response in advanced GC patients.

Albumin (Alb), an acute-phase reactant, is a protein that plays a critical role in regulating plasma oncotic pressure [6]. Fibrinogen (Fib), an acute-phase protein produced by the liver, is an important protein during the coagulation process, and it can aggregate in tumor sites [7]. Alb/Fib ratio (AFR), which combines Alb and Fib, has been widely used as a prognostic factor for several types of human malignancies [8]. This study aimed to investigate the potential ability of AFR in predicting the clinical response to chemotherapy and prognosis in GC patients.

## Materials and methods

### Patients

This was a retrospective, observational study which was approved by the Medical Institutional Ethics Committee of the researcher’s hospital (No. KY 201,408,701). This study was conducted in accordance with the Declaration of Helsinki. Eligible patients with advanced GC between 2014 and 2020 were consecutively included in this study. Inclusion criteria: (1) confirmed stage II or III GC with histological evidence; (2) scheduled to undergo postoperative chemotherapy following radical gastrectomy (D2, R0 resection); (3) Eastern Cooperative Oncology Group (ECOG) performance status [9] 0 or 1; (4) received at least 3 cycles of 5- fluorouracil (5-FU) based adjuvant chemotherapy; and (5) life expectancy ≥ 3 months. Exclusion criteria: (1) with stage I or IV GC or combined with other tumors; (2) with preoperative adjuvant treatment or postoperative radiotherapy; (3) with an autoimmune disease requiring systemic immunosuppressive treatment; (4) with the conditions affecting Alb and Fib expressions (e.g., hepatic dysfunction, hemopathy); (5) with contraindications to chemotherapy or could not tolerate chemotherapy due to the side effects or other reasons, (6) signet ring cell carcinoma or endocrine cell carcinoma, and (7) with incomplete data or loss to follow-up.

All enrolled patients received S-1 plus oxaliplatin (SOX) or capecitabine plus oxaliplatin (XELOX) chemotherapy regimens following the procedures by previous reports [10, 11]. Briefly, the SOX regimen involves 3-week cycles of 130 mg/m² intravenous oxaliplatin on day 1, oral S-1 based on the body surface area (BSA) (< 1.25 m², 80 mg daily; 1.25–1.5 m², 100 mg daily; ≥1.5 m², 120 mg daily) on days 1–14. The XELOX regimen involves 3-week cycles of 130 mg/m² intravenous oxaliplatin on day 1, oral capecitabine 1000 mg/ m² twice daily on days 1–14. The chemotherapy duration contains eight cycles (6 months).

### Data collection

The following data were collected: (1) demographic data including age, sex distribution, body mass index (BMI), habits of smoking and drinking; (2) clinical baseline data including American Society of Anesthesiologists (ASA) grade, comorbidities of diabetes and hypertension, ECOG status; (3) treatment-related data including types of surgery, surgical approach, operation time, chemotherapy regimens, and cycles of chemotherapy; (4) tumor-related data including tumor location, tumor size, tumor differentiation, Lauren’s classification, clinical TNM stage, pathological TNM stage, and Her-2 status; (5) laboratory tests before the adjuvant treatment including hemoglobin (Hb), white blood cell (WBC), platelet, creatinine (Cr), urea, Alb, Fib, carcinoembryonic antigen (CEA), cancer antigen 125 (CA125), CA19-9, and CA72-4. AFR was calculated by Alb divided by Fib. The diagnosis, clinical and histopathological stage of GC was confirmed according to the 8th edition of the Union for International Cancer Control/American Joint Committee (UICC/AJCC) classification [12].

### Follow-up

The follow-up was performed in inpatient and outpatient every 3 months for the first 2 years, every 6 months thereafter. The follow-up assessments included physical examination, gastroscopy, laboratory tests, and radiologic assessment by computed tomography (CT). The primary endpoint was set as the proportion of patients who achieved chemotherapeutic resistance, which was defined as the progression of GC during chemotherapy or recurrence within 6 months of completed chemotherapy [13]. The secondary endpoint was set as 5-year disease-free survival (DFS, defined as the time from surgery to tumor relapse, death, or the 5-year due date), overall survival (OS, defined as the time from the diagnosis to death, or the 5-year due date).

### Statistical analysis

All data were analyzed using GraphPad Prism 8.0 (GraphPad Inc., CA, USA) and SPSS 22.0 (SPSS Inc., Chicago, IL, USA). Mean ± standard deviation (SD), or number with proportion (n, %) was used for data presentation. Chi-square or Fisher exact test was used for categorical data analysis, while Student t or Mann Whitney U test was used for measurement data analysis. A receiver operating characteristic (ROC) curve analysis was performed to assess the predictive and optimal cut-off values of continuous variables for clinical response to chemotherapy using the Youden index method. Potential risk factors for clinical response to chemotherapy were determined with binary univariate and multivariate analyses using the “Enter” method. Potential prognostic factors for OS were determined by COX regression analysis. The association between survival and AFR level was examined using the Kaplan–Meier curve analysis with the log-rank test. A p value of < 0.05 was considered statistically significant.

## Results

**Fig. 1:**
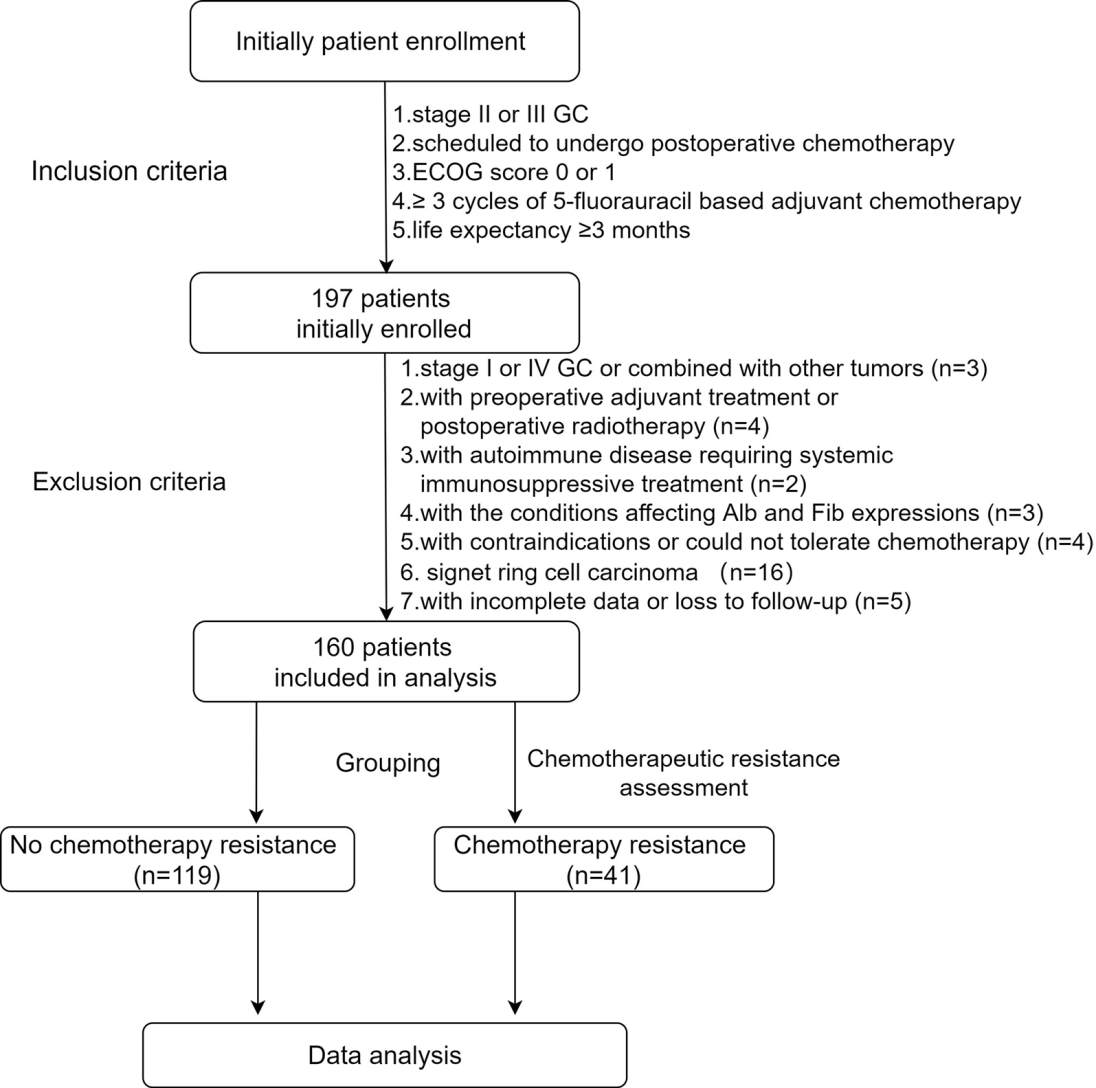
The flow chart. *GC* gastric cancer, *ECOG* Eastern Cooperative Oncology Group

There were 197 patients who met the inclusion criteria and were initially enrolled. Based on the exclusion criteria, 37 patients were then excluded (see the flow chart in Fig. 1) and a total of 160 patients were included in the final data analysis. The mean age of enrolled patients was 48.1 years, and 66.9% (107/160) of them were male patients. A total of 41 patients achieved chemotherapeutic resistance with an incidence of 25.6% (41/160). The demographic and clinical characteristics associated with chemotherapeutic resistance in advanced GC patients are summarized in Table 1. The mean age (P = 0.032) and cycles of chemotherapy (P = 0.040) were significantly lower in patients with chemotherapeutic resistance than those without chemotherapeutic resistance. In addition, patients with chemotherapeutic resistance showed significantly larger tumor sizes (P = 0.027). Additionally, current smoking habits (P = 0.013) seemed to be associated with an increased incidence of chemotherapeutic resistance. No statistical differences were observed in BMI, gender, ASA physical status, drinking habits, comorbidities of diabetes and hypertension, ECOG status, types of surgery and surgical approach, operation time, estimated blood loss, chemotherapy regimen, tumor location, tumor differentiation, Lauren’s classification, clinical and pathological TNM stage, or Her-2 status between the two groups (P > 0.05).

**Table 1:** Demographic and clinical characteristics associated with chemotherapeutic resistance in GC patients

	Chemotherapeutic resistance		
Variables	No (n = 119)	Yes (n = 41)	*P*-value
Age (year)	48.7 ± 5.4	46.5 ± 6.2	0.032^a^*
BMI (kg/m^2^)	20.9 ± 2.2	21.1 ± 2.3	0.620^a^
Gender, n (%)	–	–	0.543^c^
Male	78 (65.5)	29 (70.7)	–
Female	41 (34.5)	12 (29.3)	–
ASA physical status, n (%)	–	–	0.964^c^
I–II	75 (63.0)	26 (63.4)	–
III–IV	44 (37.0)	15 (36.6)	–
Current smoker, n (%)	19 (16.0)	14 (34.1)	0.013^c^*
Heavy drinker, n (%)	15 (12.6)	6 (14.6)	0.740^c^
Diabetes, n (%)	11 (9.2)	4 (9.8)	0.923^d^
Hypertension, n (%)	17 (14.3)	8(19.5)	0.427^c^
ECOG status, n (%)	–	–	0.515^c^
0	26 (21.8)	7 (17.1)	–
1	93 (78.2)	34 (82.9)	–
Types of surgery, n (%)	–	–	0.850^c^
Total gastrectomy	33 (27.7)	12 (29.3)	–
Partial gastrectomy	86 (72.3)	29 (70.7)	–
Surgical approach	–	–	0.416^c^
Laparotomy	37 (31.1)	10 (24.4)	–
Laparoscopic	82 (68.9)	31 (75.6)	–
Operation time (min)	225.6 ± 37.5	220.8 ± 33.7	0.470^a^
Estimated blood loss (ml)	290.5 ± 135.6	262.5 ± 145.3	0.265^b^
Chemotherapy regimen, n (%)	–	–	0.679^c^
SOX	45 (37.8)	17 (41.5)	–
XELOX	74 (62.2)	24 (58.5)	–
Cycles of chemotherapy	4.7 ± 0.8	4.4 ± 0.8	0.040^a^*
Tumor location, n (%)	–	–	0.711^c^
Upper 1/3	15 (12.6)	4 (9.8)	–
Middle 1/3	43 (36.1)	13 (31.7)	–
Low 1/3	61 (51.3)	24 (58.5)	–
Tumor size (cm)	3.5 ± 1.4	4.1 ± 1.7	0.027^b^*
Tumor differentiation, n (%)	–	–	0.417^c^
Poorly	82(68.9)	31 (75.6)	–
Moderately/well	37(31.1)	10 (24.4)	–
Lauren’s classification	–	–	0.834^c^
Intestinal	12 (10.1)	5 (12.2)	–
Diffuse	79 (66.4)	28 (68.3)	–
Mixed	28 (23.5)	8 (19.5)	–
Clinical TNM stage	–	–	0.838^c^
II	16 (13.4)	5 (12.2)	–
III	103 (86.6)	36 (87.8)	–
Pathological TNM stage	–	–	0.666^c^
II	17 (14.3)	7 (17.1)	–
III	102 (85.7)	34 (82.9)	–
Her–2	–	–	0.738^c^
Positive	17 (14.3)	5 (12.2)	–
Negative	102 (85.7)	36 (87.8)	–

*GC* gastric cancer, *BMI* body mass index, *ASA* American Society of Anesthesiologists; *ECOG* Eastern Cooperative Oncology Group, *SOX* S-1 plus oxaliplatin, *XELOX *capecitabine plus oxaliplatin. *P value < 0.05. ^a^Student t test, ^b^Mann Whitney U test, ^c^Chi-square test, ^d^Fisher exact test

The pretreatment laboratory tests associated with chemotherapeutic resistance are summarized in Table 2. In comparison with patients without chemotherapeutic resistance, patients with chemotherapeutic resistance showed significantly lower pretreatment AFR levels (P = 0.001). Additionally, those patients with elevated pretreatment CEA (P = 0.029) and CA19-9 (P = 0.034) levels were more likely to experience chemotherapeutic resistance. The laboratory tests of patients with or without chemotherapeutic resistance were not significantly different in terms of Hb, WBC, platelet, Cr, urea, CA72-4, and CA125 (P > 0.05).

–

**Table 2:** Preoperative laboratory tests associated with chemotherapeutic resistance in GC patients

	Chemotherapeutic resistance		
Patient characteristics	No (n = 119)	Yes (n = 41)	*P*-value
Hb (mg/dL)	13.0 ± 1.8	12.9 ± 1.9	0.763^a^
WBC (×10^9^/L)	7.5 ± 2.2	7.4 ± 1.9	0.796^a^
Platelet (×10^9^/L)	199.5 ± 32.4	191.1 ± 30.3	0.148^b^
Cr (mg/dL)	0.84 ± 0.13	0.81 ± 0.13	0.204^a^
Urea(mmol/L)	6.4 ± 1.2	6.5 ± 1.4	0.660^a^
AFR	11.8 ± 2.2	10.7 ± 2.0	0.001^a^*
CEA (ng/ml)	–	–	0.029^c^*
≥ 5.0	7 (5.9)	7 (17.1)	–
< 5.0	112 ( 94.1)	34 (82.9)	–
CA19–9 (kU/L)	–	–	0.034^c^*
≥ 40	11 (9.2)	9 (22.0)	–
< 40	108 (91.8)	32 (78.0)	–
CA72–4 (U/mL)	–	–	0.525^d^
≥ 6	8 (6.7)	4 (9.8)	–
< 6	111 (93.3)	37 (90.2)	–
CA125 (U/ml)	–	–	0.437^d^
≥ 35	6 (5.0)	4 (9.8)	
< 35	113 (95.0)	45 (90.2)	–

*Hb* hemoglobin, *WBC* white blood cell, *Cr* creatinine, *AFR* albumin/fibrinogen ratio, *CEA* carcinoembryonic antigen, *CA* cancer antigen. *P value < 0.05. ^a^Student *t* test, ^b^Mann Whitney *U* test, ^c^Chi-square test, ^d^Fisher exact test

**Fig. 2:**
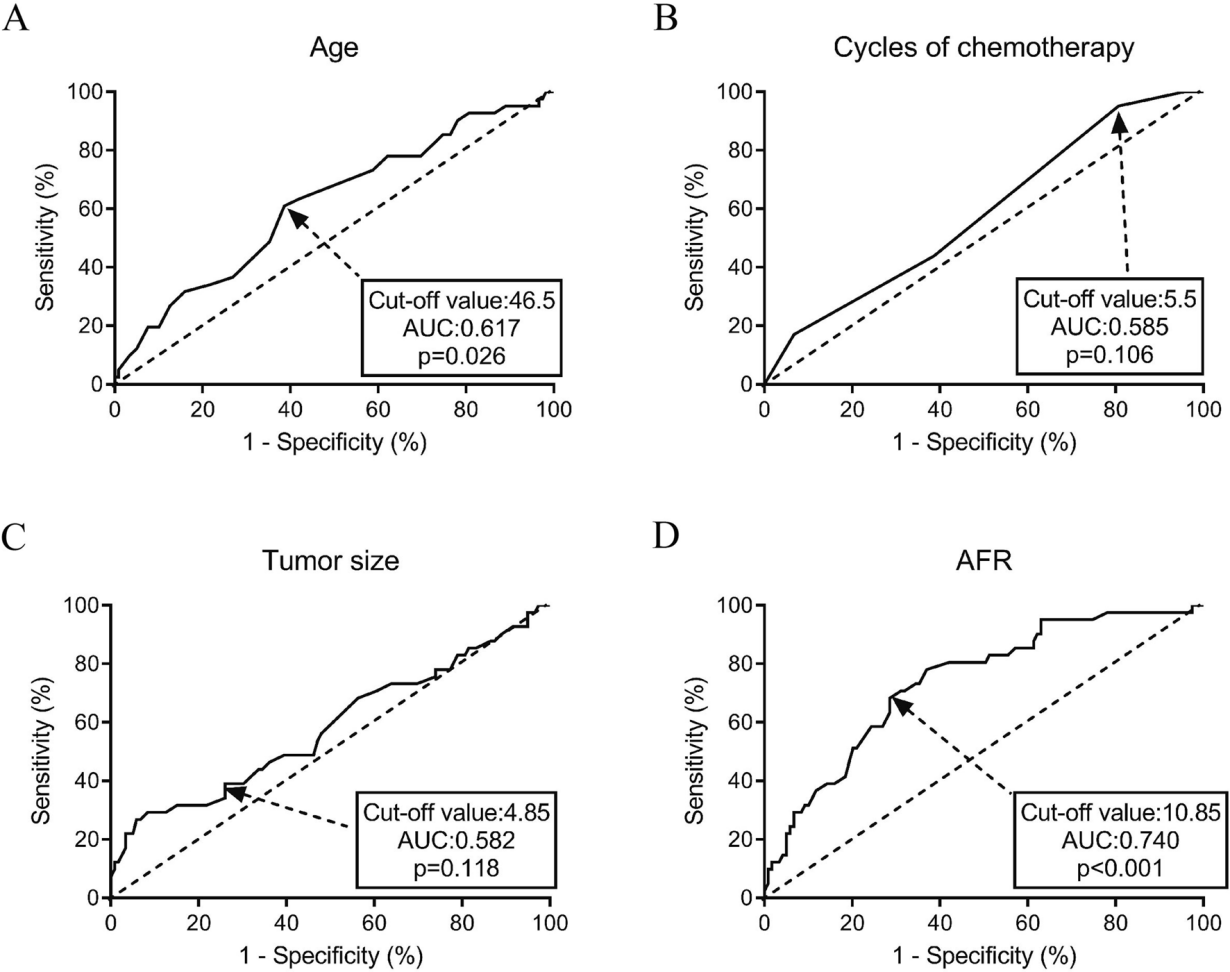
Predictors for chemotherapy resistance in GC patients by ROC curve analyses. **A** Age; **B** Cycles of chemotherapy; **C** Tumor size; **D** AFR * GC* gastric cancer; *ROC* receiver operating characteristic, *AFR* albumin/fibrinogen ratio, *AUC* area under the curve

ROC curves were constructed to evaluate the performance of four continuous variables (age, cycles of chemotherapy, tumor size, and AFR) to predict chemotherapeutic resistance (see Fig. 2). Age (cut-off value: 46.5, AUC: 0.617, P = 0.026) and AFR (cut-off value: 10.85, AUC: 0.740, P < 0.001) were two predictors of chemotherapeutic resistance by ROC curve analysis. Based on the cut-off values, these were categorized into high (≥ cut off value) and low (< cut off value) groups.

**Fig. 3:**
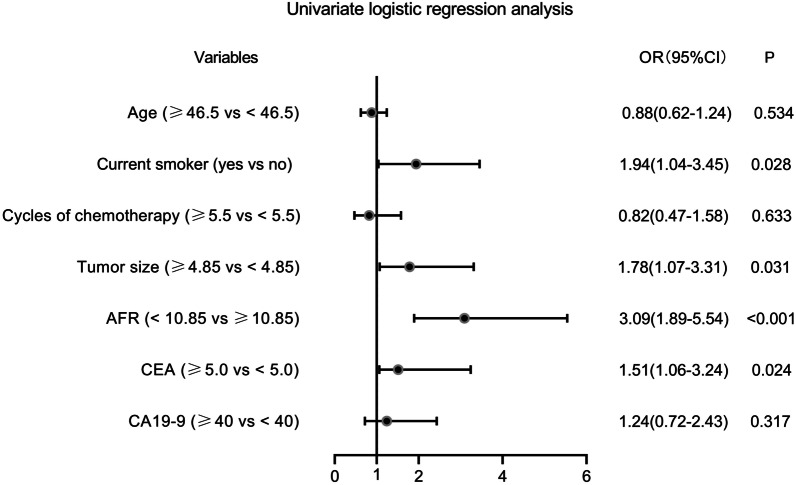
The univariate logistic regression analysis for chemotherapy resistance by forest plot. *AFR* albumin/fibrinogen ratio, *CEA* carcinoembryonic antigen, *CA* cancer antigen, *OR* odds ratio, *CI* confidence interval

**Fig. 4:**
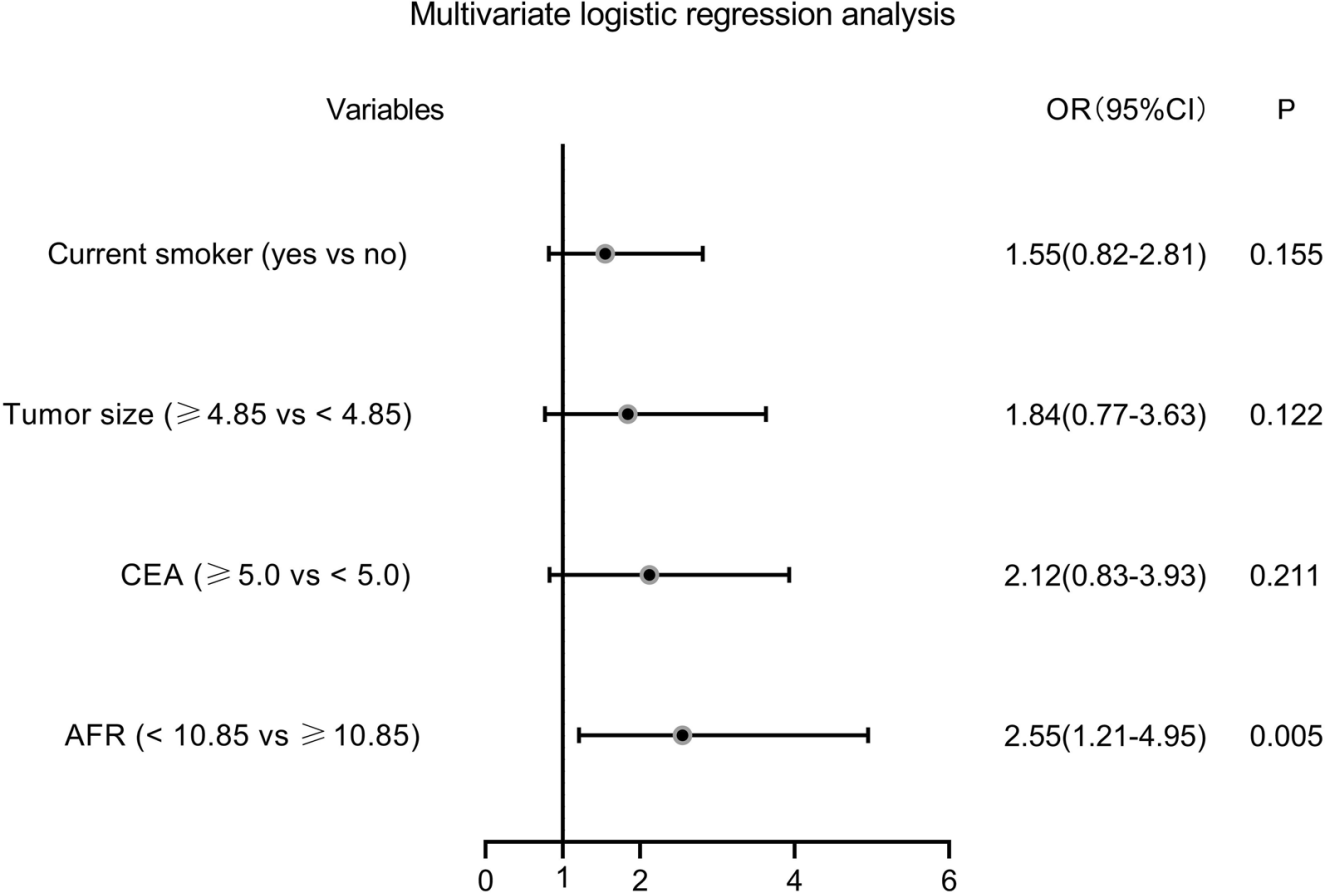
The multivariate logistic regression analysis for chemotherapy resistance by forest plot. *AFR* albumin/fibrinogen ratio, *CEA* carcinoembryonic antigen, *OR* odds ratio, *CI* confidence interval

Thereafter, seven potential risk factors (P < 0.05 in Tables 1 and 2) were included in the univariate logistic regression model. As indicated in Fig. 3, current smoking habits, large tumor size, low AFR, and high CEA were four potential risk factors for chemotherapeutic resistance. After including these four factors in the multivariate logistic regression model, the results revealed that low AFR (OR: 2.55, 95%CI: 1.21–4.95, P = 0.005) was an independent risk factor of chemotherapeutic resistance (Fig. 4). In addition, the results from multivariate COX regression analyses also indicated low AFR as an independent prognostic factor for 5-year OS (HR: 0.36, 95%CI: 0.15–0.73, P = 0.011, see Table 3).

**Table 3:** Univariate and multivariate Cox regression analyses of 5-year OS

	Univariate Multivariate				
Variables	HR (95%CI)	*P* value		HR (95%CI)	*P* value
Age (≥ 48 vs. < 48)	1.18 (0.48–2.90)	0.684			
Cycles of chemotherapy (≥ 3 vs. < 3)	1.47 (1.06–2.17)	0.041		1.34(0.65–3.27)	0.341
Tumor size (≥ 4.85 vs. < 4.85)	0.65 (0.23–1.74)	0.403			
Tumor differentiation (moderately/well vs. poor)	2.26 (1.03–5.03)	0.008		2.07 (1.11–4.01)	0.022
Clinical TNM stage (III vs. II)	0.44 (0.22–0.91)	0.013		0.28 (0.09–0.81)	0.029
AFR (< 10.85 vs. ≥ 10.85)	0.18 (0.05–0.53)	0.002		0.36 (0.15–0.73)	0.011
CEA (≥ 5.0 vs. < 5.0)	1.24 (0.45–3.43)	0.652			
CA19–9 (≥ 40 vs. < 40)	1.14 (0.48–2.81)	0.776			

*OS* overall survival, *AFR* albumin/fibrinogen ratio, *CEA *carcinoembryonic antigen, *CA* cancer antigen, *HR* hazard ratio, *CI* confidence interval

**Fig. 5:**
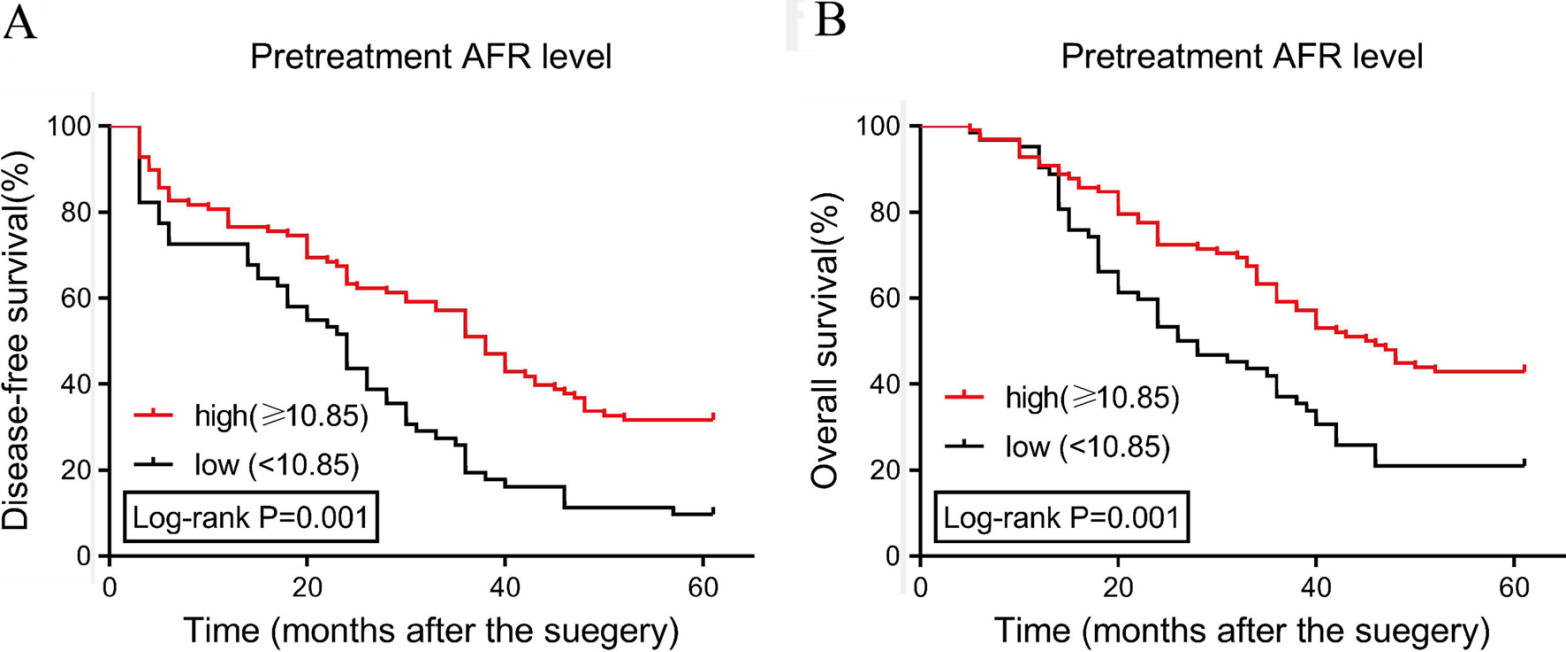
The association between pretreatment and 5-year disease-free survival **A** and overall survival **B** by Kaplan–Meier curve analysis. *AFR* albumin/fibrinogen ratio

Moreover, we performed Kaplan–Meier curve analysis to evaluate the association between the survival and pretreatment AFR level (see Fig. 5). The study’s results indicated that a low AFR (< 10.85) was associated with a poorer 5-year DFS (Fig. 5A) and OS (Fig. 5B).

## Discussion

In this study, the potential risk factors for chemotherapy resistance in GC patients were evaluated. The overall incidence of chemotherapy resistance was calculated to be 25.6%, which was quite similar to the reported 22.4% by Wan et al. [13]. The multivariate analysis indicated that low AFR was an independent risk factor for chemotherapeutic resistance in GC patients. Moreover, patients with a low AFR (< 10.85) tended to have worse clinical outcomes as determined by survival analysis. To our knowledge, this study is the first to highlight the close association between pretreatment AFR, chemotherapeutic resistance, and clinical outcomes. The close association between chemotherapy resistance and prognosis has been widely accepted [14].

It has been previously reported that preoperative AFR was an independent predictor of chemotherapy resistance and prognosis among patients with advanced epithelial ovarian cancer [14]. Additionally, a recent study by Li et al. [15] also suggested the pretreatment AFR level as a novel predictor of chemotherapy response and prognosis in patients with locally advanced rectal cancer after surgery. These findings are in agreement with our results. All these results strongly suggest a close association between AFR and chemotherapy response. A previous study by Zhang et al. [16] indicated the prognostic value of fibrinogen/pre-Albumin ratio (FPR) in patients with surgical stage II and III GC. Although with some differences (e.g. inclusion criteria, biomarkers, and observation endpoints), our study was in accordance with their conclusions.

A meta-analysis by Ma et al. [17], which included six studies, concluded that decreased lymphocyte to monocyte ratio (LMR) was associated with worse OS in GC patients. A previous study indicated that elevated neutrophil to lymphocyte ratio (NLR) correlated with late-stage GC and worse prognosis [18]. Another study suggested that preoperative, postoperative, and changes of NLR levels were all significant prognostic factors in GC patients [19]. Platelet to lymphocyte ratio (PLR), another peripheral blood-derived inflammation marker, has also been reported to be a prognostic factor in advanced GC patients after radical resection [20, 21]. In addition, the combination of NLR and PLR was also recognized as a promising predictor for tumor response and prognosis in advanced GC patients [22, 23]. Dysregulated noncoding RNAs are also involved in the mechanisms of chemotherapy resistance and have the potential to serve as novel therapeutic targets and prognostic biomarkers for GC [24, 25].

It is widely accepted that inflammation is an important contributor to the angiogenesis, proliferation, metastasis, and resistance to hormonal treatments and chemotherapy of tumors [26]. In addition, chemotherapy-induced inflammation is commonly observed in the therapeutic process, and it can lead to tumor-acquired resistance, which often results in treatment failure and tumor metastasis [27]. In patients with cancer, the expression of serum Alb is correlated with an increased tumor-induced inflammatory reaction [28]. Fib can be synthesized by tumor cells [29], and its synthesis can significantly increase in response to ongoing tumor-induced inflammation [30]. Covering both Alb and Fib, AFR can reflect systemic inflammation in patients with amplified sensitivity and effectiveness [14]. Given the very close association between inflammation and chemotherapy resistance, the predictive role of AFR in chemotherapy resistance can be understood and explained. This study indicated pretreatment AFR level as a prognostic factor in 5-year DFS and OS among GC patients. Considering the correlation between prognosis and chemotherapy resistance, the predictive role of AFR for chemotherapy resistance might be a possible explanation for its predictive role of prognosis in GC patients.

In conclusion, this study indicated that a low level of pretreatment AFR could serve as an independent predictor of chemotherapy resistance and postoperative prognosis in GC patients following radical gastrectomy. This study has some limitations. First, this is a retrospective study with relative small sample size. Second, whether pre-surgical AFR can serve as an independent predictor remains unknown. Third, the cut-off value of AFR was calculated based on our own variables and whether it can be generalized needs to be further external validated.

## Data Availability

The datasets are available from the corresponding author on reasonable request.
